# Supplementary material for: Adolescent to young adult longitudinal development of subcortical volumes in two European sites with four waves
Source: Hum Brain Mapp. 2024 Feb 24;45(3):e26574. doi: 10.1002/hbm.26574 (PMC10893970; doi:10.1002/hbm.26574)
Supplement: Supplementary file 1 — TABLE S1. Details on number of sites, total scans, age range, sample size, and scans per participant. We included all longitudinal studies of subcortical trajectories across adolescence cited in this manuscript and the present sample. TABLE S2. Quality control flow and exclusions. In post processing final data sets and (exclusions) per subcortical structure are listed. TABLE S3. Correlations between left and right hemisphere per region of interest for males and females in each wave. TABLE S4. AIC and BIC values for each structure for models 1–3. FIGURE S1. Data points for subcortical ROIs across the two sites Dresden and Paris. [file HBM-45-e26574-s001.docx]

**Supplemental material**

**S1 Introduction**

Regarding sampling type, most studies used accelerated longitudinal designs with participants starting at different ages and thus contributing limited data to the age-range of interest (Herting et al., 2018; Narvacan et al., 2017; Raznahan et al., 2014; L. M. Wierenga et al., 2018). These designs are limited because age effects are confounded with interactions between a given starting age and when the measurement happened and participants only contribute data to parts of the time span investigated. Some studies included differing sex ratios across ages (Herting et al., 2018) or did not report detailed sociodemographic sample description like IQ or socioeconomic status (Herting et al., 2018; Tamnes 2013) limiting understanding of the generalizability of findings. Image acquisition differed in field strengths with most studies using 1.5 Tesla (except Wierenga et al. (2018) using 3 Tesla). Differences in quality control procedures may have also impacted the results (Backhausen et al., 2016; Ducharme et al., 2016). The type of segmentation software can also influence volume estimates (Makowski et al., 2018; Morey et al., 2010), even across different versions of the same software (e.g. differences in putamen using FreeSurfer 5.3 versus 6.0, see release notes). Only in some analysis software packages a longitudinal processing approach is included (see Vijayakumar et al., 2018 for a software overview) and thus has not always been used.

**Table S1.** Details on number of sites, total scans, age range, sample size and scans per participant. We included all longitudinal studies of subcortical trajectories across adolescence cited in this manuscript and the present sample.

| **Studies** | **N sites** | **N scans** | **Age (y)** | **N** | **Scans per participant** |
| --- | --- | --- | --- | --- | --- |
| Backhausen et al., 2023  (this manuscript) | 2 | 1408 | 14 to 24 | 503 | up to 4 (1 scan: 76, 2 scans: 98, 3 scans: 178, 4 scans: 151) |
|  |  |  |  |  |  |
| Dennison et al., 2013 | 1 | 120 | 17 to 17 | 60 | 2 |
| Goddings et al., 2014 | 1 | 711 | 7 to 19 | 275 | 2 or more |
| Herting et al., 2018 | 3 | 467 | 10 to 22 | 216 | up to 3 (1 scan: 251, 2 scans: 181, 3 scans: 35) |
| Koolschijn & Crone, 2013 | 1 | 442 | 8 to 30 | 442 | 1 |
| Lenroot et al., 2007 | 1 | 829 | 3 to 27 | 387 | up to 7 (1 scan: 159, 2 scans: 103, 3 scans: 59, 4 scans: 47, 5 scans: 16, 6 scans: 2, 7 scans: 1) |
| Narvacan et al., 2017 | 1 | 168 | 5 to 27 | 84 | 2 |
| Østby et al., 2009 | 1 | 316 | 8 to 30 | 171 | 1 scan: 26, or 2 scans: 145 |
| Raznahan et al., 2014 | 1 | 1172 | 5 to 25 | 618 | 1 scan: 278, 2 scans: 192, 3 or more scans: 148 |
| Sowell et al., 2002 | 1 | 105 | 7 to 16 | 35 | 3 |
| Tamnes et al., 2013 | 1 | 170 | 8 to 22 | 85 | 2 |
| Wierenga et al., 2014 | 1 | 223 | 7 to 24 | 147 | up to 4 (1 scan: 170, 2 to 4 scans: 53) |
| Wierenga et al., 2018 | 9 | n.a. | 3 to 21 | 1234 | up to 3 |
| Wierenga et al., 2018 | 1 | 680 | 8 to 29 | 271 | up to 3 (1 scan: 30, 2 scans: 73, 3 scans: 168) |

**Table S2.** Quality control flow and exclusions. In post processing final data sets and (exclusions) per subcortical structure are listed.

We excluded single subcortical structures when segmented incorrectly. If more than two structures were affected in one image we excluded all structures from quantitative analyses.

**DRESDEN**

| **Pre processing** | **WAVE 1** | **WAVE 2** | **WAVE 3** | **WAVE 4** |
| --- | --- | --- | --- | --- |
| Total MPRAGE scans available | 232 | 218 | 180 | 138 |
| Pre processing QC fail | 30 | 11 | 7 | 8 |
| Available for FS processing | 202 | 207 | 173 | 130 |
| Successfully processed | 202 | 207 | 173 | 130 |
| Post processing QC fail | 1 | 0 | 1 | 0 |
| Total QC fail | 31 | 11 | 8 | 8 |
| Total QC fail percentage | 13.36% | 5.05% | 4.44% | 5.80% |
| Final number of data sets | 201 | 207 | 172 | 130 |

| **Post processing** | **WAVE 1** | **WAVE 2** | **WAVE 3** | **WAVE 4** |
| --- | --- | --- | --- | --- |
| Thalamus | 199 (3) | 207 (0) | 172 (1) | 130 (0) |
| Globus pallidus | 200 (2) | 204 (3) | 172 (1) | 129 (1) |
| Caudate nucleus | 199 (3) | 207 (0) | 172 (1) | 130 (0) |
| Putamen | 200 (2) | 204 (3) | 172 (1) | 129 (1) |
| Nucleus accumbens | 201 (1) | 207 (0) | 172 (1) | 130 (0) |
| Hippocampus | 198 (4) | 206 (1) | 172 (1) | 130 (0) |
| Amygdala | 200 (2) | 206 (1) | 172 (1) | 130 (0) |

**PARIS**

| **Pre processing** | **WAVE 1** | **WAVE 2** | **WAVE 3** | **WAVE 4** |
| --- | --- | --- | --- | --- |
| Total MPRAGE scans available | 257 | 138 | 205 | 123 |
| Pre processing QC fail | 11 | 8 | 4 | 0 |
| Available for FS processing | 246 | 130 | 201 | 123 |
| Successfully processed | 246 | 130 | 201 | 123 |
| Post processing QC fail | 0 | 1 | 1 | 0 |
| Total QC fail | 11 | 9 | 5 | 0 |
| Total QC fail percentage | 4.28% | 6.52% | 2.44% | 0.00% |
| Final number of data sets | 246 | 129 | 200 | 123 |

| **Post processing** | **WAVE 1** | **WAVE 2** | **WAVE 3** | **WAVE 4** |
| --- | --- | --- | --- | --- |
| Thalamus | 246 (0) | 129 (1) | 200 (1) | 123 (0) |
| Globus pallidus | 246 (0) | 129 (1) | 200 (1) | 123 (0) |
| Caudate nucleus | 246 (0) | 129 (1) | 199 (2) | 123 (0) |
| Putamen | 246 (0) | 129 (1) | 200 (1) | 123 (0) |
| Nucleus accumbens | 246 (0) | 129 (1) | 200 (1) | 123 (0) |
| Hippocampus | 245 (1) | 127 (3) | 199 (2) | 123 (0) |
| Amygdala | 245 (1) | 129 (1) | 200 (1) | 123 (0) |

**Table S3**. Correlations between left and right hemisphere per region of interest for males and females in each wave.

|  | Dresden | |  | Paris | |
| --- | --- | --- | --- | --- | --- |
|  | *r* | *p* |  | *r* | *p* |
| *Thalamus* |  |  |  |  |  |
| Wave 1 – female | 0.83 | *** |  | 0.92 | *** |
| Wave 1 - male | 0.86 | *** |  | 0.91 | *** |
| Wave 2 - female | 0.85 | *** |  | 0.92 | *** |
| Wave 2 - male | 0.89 | *** |  | 0.91 | *** |
| Wave 3- female | 0.83 | *** |  | 0.91 | *** |
| Wave 3 - male | 0.84 | *** |  | 0.86 | *** |
| Wave 4 - female | 0.87 | *** |  | 0.93 | *** |
| Wave 4 - male | 0.89 | *** |  | 0.93 | *** |
|  |  |  |  |  |  |
| *Globus pallidus* |  |  |  |  |  |
| Wave 1 – female | 0.80 | *** |  | 0.85 | *** |
| Wave 1 - male | 0.72 | *** |  | 0.84 | *** |
| Wave 2 - female | 0.71 | *** |  | 0.87 | *** |
| Wave 2 - male | 0.76 | *** |  | 0.90 | *** |
| Wave 3- female | 0.78 | *** |  | 0.80 | *** |
| Wave 3 - male | 0.78 | *** |  | 0.87 | *** |
| Wave 4 - female | 0.77 | *** |  | 0.83 | *** |
| Wave 4 - male | 0.80 | *** |  | 0.86 | *** |
|  |  |  |  |  |  |
| *Caudate nucleus* |  |  |  |  |  |
| Wave 1 – female | 0.94 | *** |  | 0.95 | *** |
| Wave 1 - male | 0.92 | *** |  | 0.95 | *** |
| Wave 2 - female | 0.94 | *** |  | 0.95 | *** |
| Wave 2 - male | 0.92 | *** |  | 0.96 | *** |
| Wave 3- female | 0.94 | *** |  | 0.94 | *** |
| Wave 3 - male | 0.94 | *** |  | 0.95 | *** |
| Wave 4 - female | 0.94 | *** |  | 0.94 | *** |
| Wave 4 - male | 0.94 | *** |  | 0.96 | *** |
|  |  |  |  |  |  |
| *Putamen* |  |  |  |  |  |
| Wave 1 – female | 0.89 | *** |  | 0.96 | *** |
| Wave 1 - male | 0.91 | *** |  | 0.97 | *** |
| Wave 2 - female | 0.91 | *** |  | 0.96 | *** |
| Wave 2 - male | 0.87 | *** |  | 0.97 | *** |
| Wave 3- female | 0.88 | *** |  | 0.93 | *** |
| Wave 3 - male | 0.92 | *** |  | 0.96 | *** |
| Wave 4 - female | 0.91 | *** |  | 0.94 | *** |
| Wave 4 - male | 0.90 | *** |  | 0.97 | *** |
|  |  |  |  |  |  |
| *Nucleus accumbens* |  |  |  |  |  |
| Wave 1 – female | 0.67 | *** |  | 0.67 | *** |
| Wave 1 - male | 0.46 | *** |  | 0.73 | *** |
| Wave 2 - female | 0.68 | *** |  | 0.66 | *** |
| Wave 2 - male | 0.46 | *** |  | 0.74 | *** |
| Wave 3- female | 0.72 | *** |  | 0.65 | *** |
| Wave 3 - male | 0.53 | *** |  | 0.68 | *** |
| Wave 4 - female | 0.69 | *** |  | 0.72 | *** |
| Wave 4 - male | 0.51 | *** |  | 0.79 | *** |
|  |  |  |  |  |  |
| *Hippocampus* | 0.80 | *** |  | 0.86 | *** |
| Wave 1 – female | 0.83 | *** |  | 0.84 | *** |
| Wave 1 - male | 0.76 | *** |  | 0.90 | *** |
| Wave 2 - female | 0.83 | *** |  | 0.81 | *** |
| Wave 2 - male | 0.80 | *** |  | 0.85 | *** |
| Wave 3- female | 0.78 | *** |  | 0.86 | *** |
| Wave 3 - male | 0.81 | *** |  | 0.83 | *** |
| Wave 4 - female | 0.80 | *** |  | 0.85 | *** |
| Wave 4 - male | 0.80 | *** |  | 0.86 | *** |
|  |  |  |  |  |  |
| *Amygdala* |  |  |  |  |  |
| Wave 1 – female | 0.72 | *** |  | 0.69 | *** |
| Wave 1 - male | 0.75 | *** |  | 0.72 | *** |
| Wave 2 - female | 0.75 | *** |  | 0.76 | *** |
| Wave 2 - male | 0.77 | *** |  | 0.73 | *** |
| Wave 3- female | 0.77 | *** |  | 0.68 | *** |
| Wave 3 - male | 0.77 | *** |  | 0.71 | *** |
| Wave 4 - female | 0.74 | *** |  | 0.72 | *** |
| Wave 4 - male | 0.79 | *** |  | 0.60 | *** |

*** *P-value* <.001.

**Table S4.** AIC and BIC values for each structure for models 1 - 3.

|  | **AIC** | | | **BIC** | | |
| --- | --- | --- | --- | --- | --- | --- |
| **Structure** | Model 1 | Model 2 | Model 3 | Model 1 | Model 2 | Model 3 |
| Thalamus | 19719.76 | 19564.18 | 19541.10 | 19756.49 | 19606.16 | 19593.58 |
| Globus Pallidus | 16524.41 | 16427.87 | 16278.20 | 16561.13 | 16469.84 | 16330.66 |
| Caudate Nucleus | 18034.79 | 17984.28 | 17851.52 | 18055.78 | 18010.52 | 17903.99 |
| Putamen | 18601.97 | 18459.73 | 18412.57 | 18638.69 | 18501.70 | 18465.03 |
| Nucleus Accumbens | 14950.59 | 14906.73 | 14884.50 | 14987.33 | 14948.72 | 14937.00 |
| Hippocampus | 17797.75 | 17677.13 | 17631.97 | 17834.46 | 17719.08 | 17684.40 |
| Amygdala | 16585.26 | 16391.31 | 16325.84 | 16621.99 | 16433.28 | 16378.31 |

AIC = Akaike Information Criterion; BIC = Baysian Information Criterion.

**Figure S1.** Data points for subcortical ROIs across the two sites Dresden and Paris.


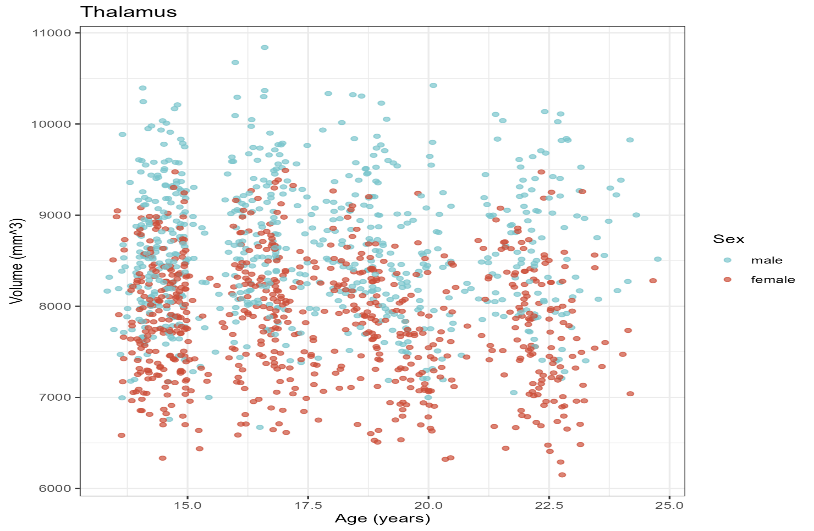

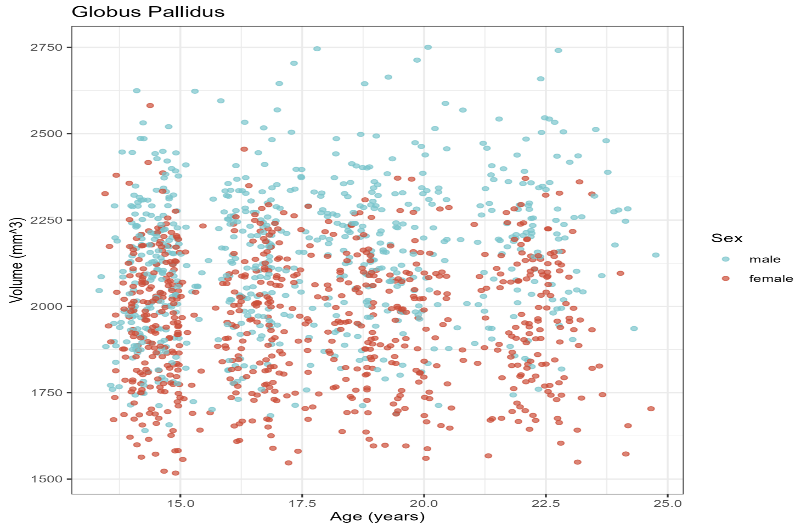

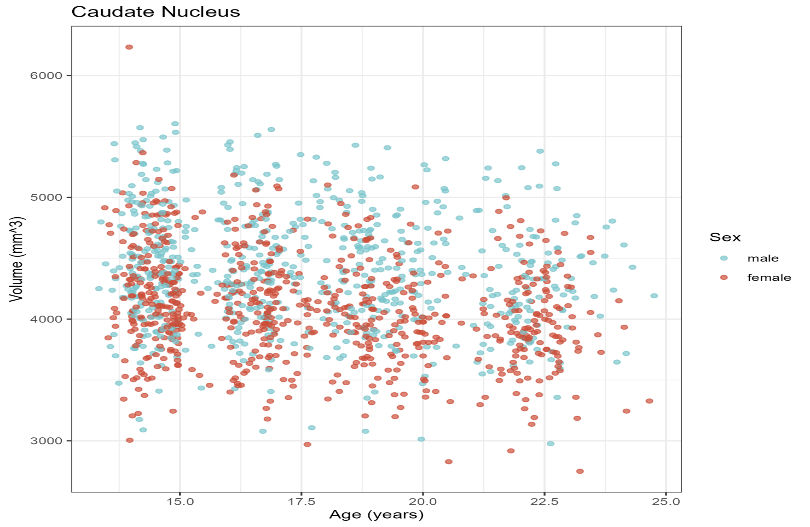

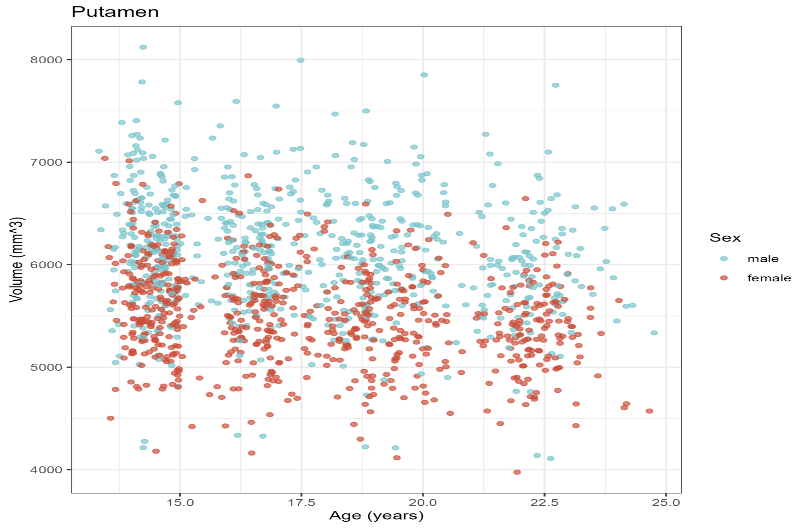

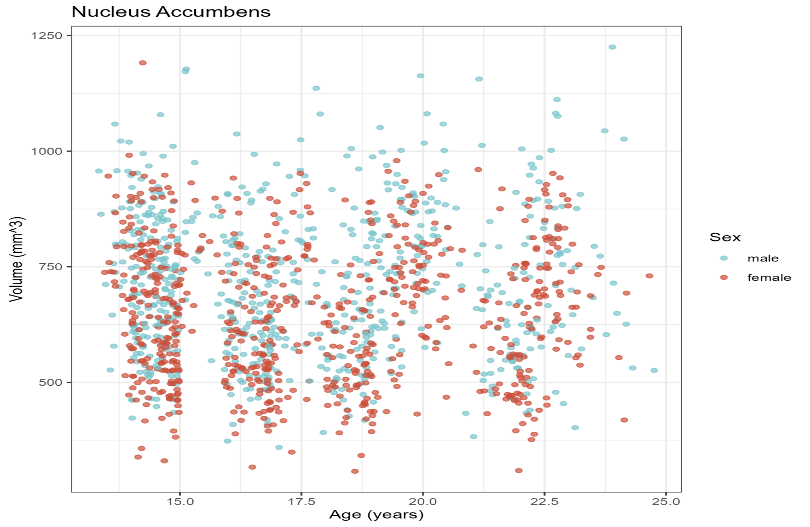

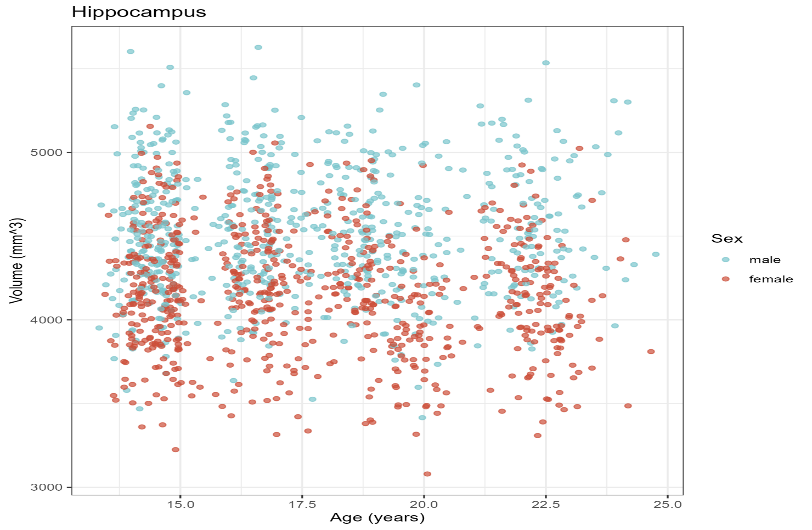

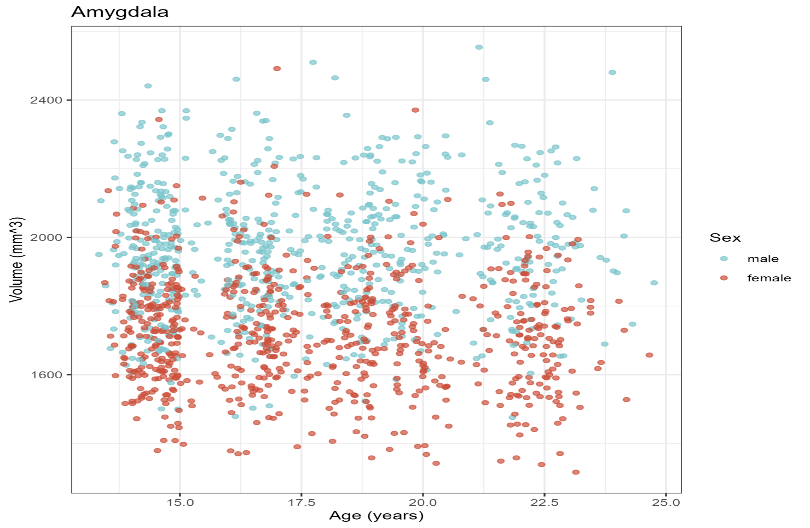

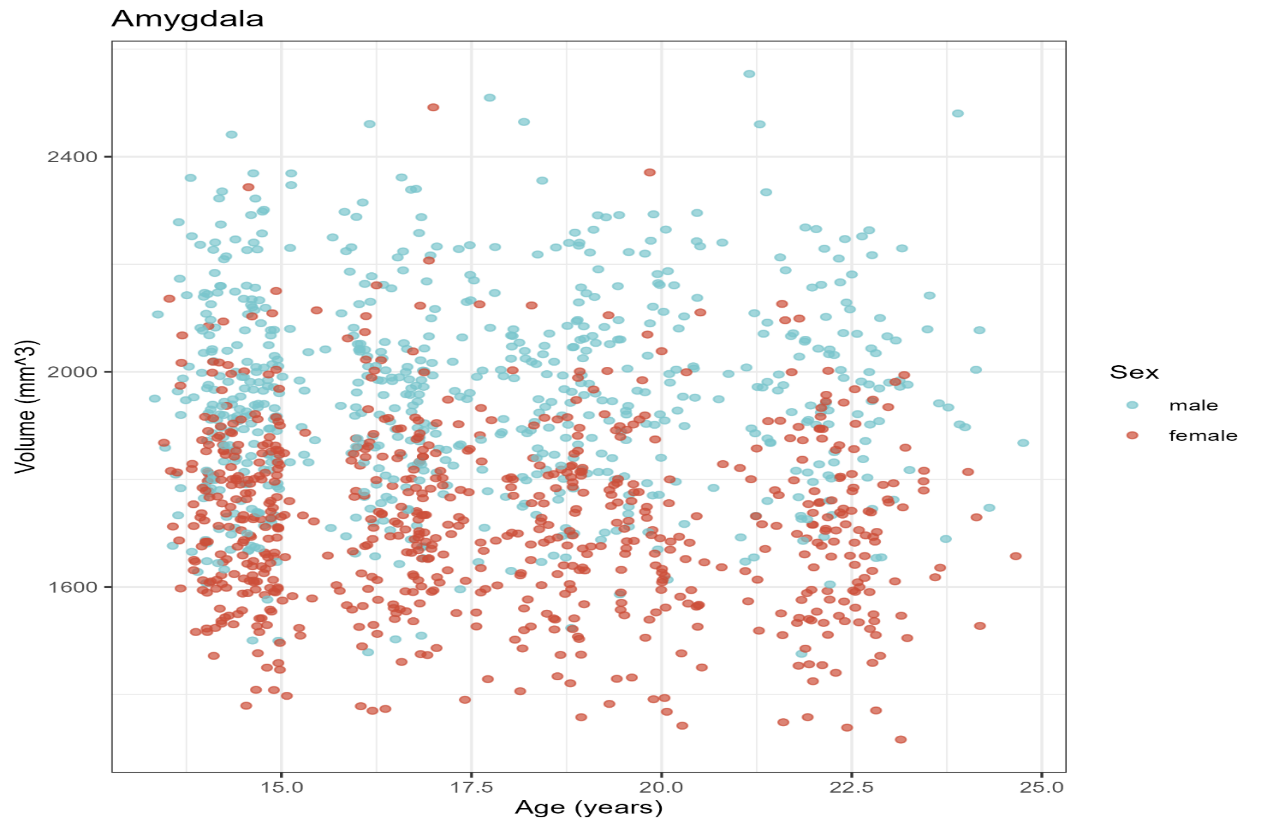


**Supplemental References**

Backhausen, L. L., Herting, M. M., Buse, J., Roessner, V., Smolka, M. N., & Vetter, N. C. (2016). Quality Control of Structural MRI Images Applied Using FreeSurfer—A Hands-On Workflow to Rate Motion Artifacts. *Frontiers in Neuroscience*, *10*. https://doi.org/10.3389/fnins.2016.00558

Dennison, M., Whittle, S., Yücel, M., Vijayakumar, N., Kline, A., Simmons, J., & Allen, N. B. (2013). Mapping subcortical brain maturation during adolescence: Evidence of hemisphere- and sex-specific longitudinal changes. *Developmental Science*, *16*(5), 772–791. https://doi.org/10.1111/desc.12057

Ducharme, S., Albaugh, M. D., Nguyen, T.-V., Hudziak, J. J., Mateos-Pérez, J. M., Labbe, A., Evans, A. C., & Karama, S. (2016). Trajectories of cortical thickness maturation in normal brain development—The importance of quality control procedures. *NeuroImage*, *125*, 267–279. https://doi.org/10.1016/j.neuroimage.2015.10.010

Goddings, A.-L., Mills, K. L., Clasen, L. S., Giedd, J. N., Viner, R. M., & Blakemore, S.-J. (2014). The influence of puberty on subcortical brain development. *NeuroImage*, *88*, 242–251. https://doi.org/10.1016/j.neuroimage.2013.09.073

Herting, M. M., Johnson, C., Mills, K. L., Vijayakumar, N., Dennison, M., Liu, C., Goddings, A.-L., Dahl, R. E., Sowell, E. R., Whittle, S., Allen, N. B., & Tamnes, C. K. (2018). Development of subcortical volumes across adolescence in males and females: A multisample study of longitudinal changes. *NeuroImage*, *172*, 194–205. https://doi.org/10.1016/j.neuroimage.2018.01.020

Koolschijn, P. C. M. P., & Crone, E. A. (2013). Sex differences and structural brain maturation from childhood to early adulthood. *Developmental Cognitive Neuroscience*, *5*, 106–118. https://doi.org/10.1016/j.dcn.2013.02.003

Lenroot, R. K., Gogtay, N., Greenstein, D. K., Wells, E. M., Wallace, G. L., Clasen, L. S., Blumenthal, J. D., Lerch, J., Zijdenbos, A. P., Evans, A. C., Thompson, P. M., & Giedd, J. N. (2007). Sexual dimorphism of brain developmental trajectories during childhood and adolescence. *NeuroImage*, *36*(4), 1065–1073. https://doi.org/10.1016/j.neuroimage.2007.03.053

Makowski, C., Béland, S., Kostopoulos, P., Bhagwat, N., Devenyi, G. A., Malla, A. K., Joober, R., Lepage, M., & Chakravarty, M. M. (2018). Evaluating accuracy of striatal, pallidal, and thalamic segmentation methods: Comparing automated approaches to manual delineation. *NeuroImage*, *170*, 182–198. https://doi.org/10.1016/j.neuroimage.2017.02.069

Morey, R. A., Selgrade, E. S., Wagner II., H. R., Huettel, S. A., Wang, L., & McCarthy, G. (2010). Scan-rescan reliability of subcortical brain volumes derived from automated segmentation. *Human Brain Mapping*, *31*(11), 1751–1762. Scopus. https://doi.org/10.1002/hbm.20973

Narvacan, K., Treit, S., Camicioli, R., Martin, W., & Beaulieu, C. (2017). Evolution of deep gray matter volume across the human lifespan. *Human Brain Mapping*, *38*(8), 3771–3790. https://doi.org/10.1002/hbm.23604

Østby, Y., Tamnes, C. K., Fjell, A. M., Westlye, L. T., Due-Tønnessen, P., & Walhovd, K. B. (2009). Heterogeneity in Subcortical Brain Development: A Structural Magnetic Resonance Imaging Study of Brain Maturation from 8 to 30 Years. *Journal of Neuroscience*, *29*(38), 11772–11782. https://doi.org/10.1523/JNEUROSCI.1242-09.2009

Raznahan, A., Shaw, P. W., Lerch, J. P., Clasen, L. S., Greenstein, D., Berman, R., Pipitone, J., Chakravarty, M. M., & Giedd, J. N. (2014). Longitudinal four-dimensional mapping of subcortical anatomy in human development. *Proceedings of the National Academy of Sciences*, *111*(4), 1592–1597. https://doi.org/10.1073/pnas.1316911111

Sowell, E. R., Trauner, D. A., Gamst, A., & Jernigan, T. L. (2002). Development of cortical and subcortical brain structures in childhood and adolescence: A structural MRI study. *Developmental Medicine & Child Neurology*, *44*(1), 4–16. https://doi.org/10.1111/j.1469-8749.2002.tb00253.x

Tamnes, C. K., Walhovd, K. B., Dale, A. M., Østby, Y., Grydeland, H., Richardson, G., Westlye, L. T., Roddey, J. C., Hagler, D. J., Due-Tønnessen, P., Holland, D., & Fjell, A. M. (2013). Brain development and aging: Overlapping and unique patterns of change. *NeuroImage*, *68*, 63–74. https://doi.org/10.1016/j.neuroimage.2012.11.039

Vijayakumar, N., Mills, K. L., Alexander-Bloch, A., Tamnes, C. K., & Whittle, S. (2018). Structural brain development: A review of methodological approaches and best practices. *Developmental Cognitive Neuroscience*, *33*, 129–148. https://doi.org/10.1016/j.dcn.2017.11.008

Wierenga, L. M., Bos, M. G. N., Schreuders, E., vd Kamp, F., Peper, J. S., Tamnes, C. K., & Crone, E. A. (2018). Unraveling age, puberty and testosterone effects on subcortical brain development across adolescence. *Psychoneuroendocrinology*, *91*, 105–114. https://doi.org/10.1016/j.psyneuen.2018.02.034

Wierenga, L. M., Langen, M., Ambrosino, S., van Dijk, S., Oranje, B., & Durston, S. (2014). Typical development of basal ganglia, hippocampus, amygdala and cerebellum from age 7 to 24. *NeuroImage*, *96*, 67–72. https://doi.org/10.1016/j.neuroimage.2014.03.072

Wierenga, L., Sexton, J. A., Laake, P., Giedd, J. N., Tamnes, C. K., & the Pediatric Imaging, N., and Genetics Study. (2018). A Key Characteristic of Sex Differences in the Developing Brain: Greater Variability in Brain Structure of Boys than Girls. *Cerebral Cortex*, *28*(8), 2741–2751. https://doi.org/10.1093/cercor/bhx154
